# Social and Behavioral Determinants for Early Childhood Caries among Preschool Children in India

**DOI:** 10.15171/joddd.2014.023

**Published:** 2015-06-10

**Authors:** Mitali Jain, Ritu Namdev, Meenakshi Bodh, Samir Dutta, Parul Singhal, Arun Kumar

**Affiliations:** ^1^Postgraduate Student, Department of Pedodontics and Preventive Dentistry, Postgraduate Institute of Dental Sciences, Rohtak, India; ^2^Associate Professor, Department of Pedodontics and Preventive Dentistry, Postgraduate Institute of Dental Sciences, Rohtak, India; ^3^Senior Professor and Head, Department of Pedodontics and Preventive Dentistry, Postgraduate Institute of Dental Sciences, Rohtak, India; ^4^Assistant Professor, Department of Pedodontics and Preventive Dentistry, Postgraduate Institute of Dental Sciences, Rohtak, India

**Keywords:** Preschool children, dental caries, questionnaire, risk factors, tooth brushing

## Abstract

***Background and aims.*** Early Childhood Caries (ECC) is a public health problem with biological, social and behavioural determinants and the notion that the principal etiology is inappropriate feeding modalities is no longer tenable. Hence this study was undertaken to assess the relationship between ECC and socio-demographic factors, dietary habits, oral hygiene habits and parental characteristics.

***Materials and methods.*** The study involved a dental examination of 1400 children aged 0-71 months, recording caries using Gruebbel’s deft index and a structured questionnaire to interview parents or caretakers. The tabulated data was statistically analyzed using t-test and ANOVA at 5% level of significance.

***Results.*** The variables significantly associated with ECC were age (P<0.001), geographical location (P<0.05), duration of breast/bottle feeding (P<0.001), use of sweetened pacifiers (P<0.001), frequency of snacking (P<0.05), frequency of tooth brushing (P<0.001), the person responsible for child’s oral health care (P<0.05) and education level of parents (P<0.05). However, other variables like child’s gender, number of siblings, types of snack the child preferred and age at which tooth brushing was instituted did not have statistically significant relationship with ECC (P>0.05).

***Conclusion.*** ECC is preventable and manageable with proper information and skills. It is important for healthcare professionals, family physicians and parents to be cognizant of the involved risk factors as their preventive efforts represent the first line of defense.

## Introduction

Dental caries is a multifactorial disease that starts with microbiological shifts within the complex biofilm and is affected by salivary flow and composition, exposure to fluoride, consumption of dietary sugars, etc.^1^Dental caries affects humans of all ages all over the world and remains a major dental health problem among schoolchildren globally.^2^ It is a disease that can never be eradicated because of the complex interaction of cultural, social, behavioral, nutritional and biological risk factors that are associated with its initiation and progression.^3^

Childhood and early adolescence are crucial periods in the development of healthy dentition. ECC is a major public health problem, being the most common chronic infectious childhood disease, which is difficult to control. While not life-threatening, its impact on individuals and communities is considerable, resulting in pain, impairment of function, deleterious influence on the child’s growth rate, body weight, and ability to thrive, thus reducing quality of life.^4^

Caries in infants and young children has long been recognized as a clinical syndrome, described by Belterami^5^ in 1930s as “Les dents noire de tout-petits” which means “black teeth of the very young.” Fass is perhaps the most popular in this perspective for defining the term “nursing bottle mouth”.^6^

Subsequently, other terms such as “baby bottle tooth decay”, “nursing bottle syndrome”, “bottle mouth caries”, “nursing caries”, “rampant caries”, “nursing bottle mouth”, “milk bottle syndrome”, “breast milk tooth decay” and “facio-lingual pattern of decay” have also been used to describe this condition.^7^

ECC was historically attributed to inappropriate and prolonged bottle use or breastfeeding. The use of the bottle, especially at bedtime, is believed to be associated with increased risk for caries, but this might not be the only factor in caries development in early childhood. The associated risk factors have also been found to vary from population to population.^8^ Moreover, the associations between ECC and improper feeding modalities are not consistent,^9,10^ and they are no longer considered to be the principal etiology. Fruit juices and carbonated beverages have also been implicated in children diagnosed with ECC.

Some studies have reported that a child’s brushing habit and frequency of brushing are also associated with the occurrence and development of dental caries.^11,12^ The education level of parents has been shown to be co-related with the occurrence and severity of ECC in their children. Lower prevalence of dental caries have been associated with higher levels of parental education.^13^ ECC is more common in children from single-parent families and those with parents of low educational level, especially of illiterate mothers.^14^

The universal prevalence of dental disease is a constant reminder of the need for effective preventive dental health education. Intervention during early childhood would seem to be the most appropriate action to ensure healthy dental habits throughout life. For the implementation of preventive attributes in a given population, knowledge about the existing standards of health and existing practices and attributes of that particular population is essential.

Hence this study aimed to assess the associations between early childhood caries and child’s age, gender, feeding habits, brushing habits, dietary habits and socioeconomic status of parents so that the physical, psychological and economic consequences of ECC can be avoided.

## Materials and Methods

The study sample comprised of 1400 preschool children age 71 months or younger with early childhood caries reporting to the Outpatient Department of Pedodontics and Preventive Dentistry, Postgraduate Institute of Dental Sciences, Rohtak, Haryana (India), during the study period. Consent was obtained for dental examination of the child from the concerned guardian to cooperate with the examiner explaining the importance of the study. The children with any systemic or any congenital syndromes or suffering from any skeletal and dental developmental disorders were excluded from the study.

The accompanying guardians were first interviewed with a self-designed, structured questionnaire (see Additional file 1). The questionnaire consisted of 5 parts. Part I included questions on general information (age, sex, address, number of siblings) about the children; in parts II and III feeding and dietary habits of the children were assessed. Parts IV and V of the questionnaire determined the oral hygiene habits and socioeconomic status of the children, respectively. Prior to being finalized, the questionnaire was pilot-tested on a group of 100 parents to ensure validity and reliability. The questionnaire’s reliability was checked using SPSS, and Cronbach’s alpha coefficient was estimated at 0.79. The interviewer himself recorded the answers to the questions in order to minimize misinterpretation of questions and to ensure uniformity in data.

The dental examination was carried out in the clinic with the patient sitting comfortably in dental chair or using the knee-to-knee examination method in case of very young children. All the primary teeth were examined using a mouth mirror and a dental explorer. Diagnosis depended upon visual evidence of lesion. The explorer was used only to remove food debris and no attempt was made to use an explorer to confirm catch of the lesions. No radiographs were taken for any child. Sterilized instruments and separate gloves were used for each patient. Caries status of each child was recorded using ‘def’ index as described by Gruebbel in 1944.^15^

As defined by Gruebbel:

d = decayed teeth (evidence of dental caries, cavitation, including filled teeth with recurrent caries),

e = extracted teeth and decayed teeth indicated for extraction due to caries,

f = filled (restored teeth without recurrent caries).

Calculation of def index: Total d + e + f = def

Data management and statistical analysis was carried out using SPSS 19 to derive the results and draw conclusion. The statistical tests used were t-test for significance of differences in mean values between the two groups and ANOVA for significance of differences in mean values between more than two groups. Statistical significance was set at P<0.05.

## Results

The study sample included 1400 children aged ≤71 months. Caries status of each child was recorded using ‘def’ index as described by Gruebbel in 1944.

**Figure 1.**
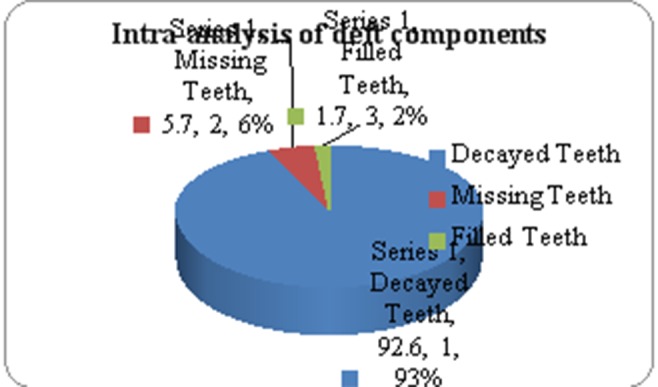
Intra-analysis of deft components

The decayed teeth (92.6%) constituted the major component of deft, with missing teeth contributing to only 5.7% and filled teeth to only 1.7 % of deft (Figure 1).

As shown in Table 1, the mean deft increased with age and differences in mean deft between different age groups were found to be highly significant statistically (P<0.001). The study sample included 686 (49%) male and 714 (51%) female children, with no significant differences in deft between male and female children (P>0.05). The mean deft was higher (5.19±2.909) in children from rural areas compared to those from urban areas (4.66±2.113). No significant association was found between deft and the number of siblings (P>0.05).

**Table 1:** Demographic characteristics and ECC

		**Number of children**	**%**	**Average deft**	**Standard deviation**	**P**
1	**Age**	0-12 mos	0	0	0	0	P<0.001
		12-23 mos	14	1	1	0.00	
		24-35 mos	140	10	2.1	0.703	
		36-47 mos	308	22	3.72	0.864	
		48-59 mos	490	35	4.74	1.156	
		60-71 mos	448	32	4.88	3.10
2	**Gender**	Male	686	49	4.92	2.844	P>0.05
		Female	714	51	4.84	2.082	
3	**Geographical location**	Rural	574	41	5.19	2.909	P<0.05
	Urban	826	59	4.66	2.113	
4	**Number of siblings**	0	154	11	4.81	2.13	P>0.05
		1	672	48	5.08	2.50	
		2	490	35	4.74	2.625	
		3 or more	84	6	4.16	1.875	

Table 2summarizes the association of ECC with feeding habits of children. The average deft was lowest (4.65±1.996) in children who were breast-fed only up to 1 year of age and highest (5.2±2.978) in children who were breast-fed for more than 2 years and this difference in deft was found to be statistically significant (P<0.05). Of 1400 children, only 28 (2%) children were not bottle-fed, 784 (56%) children were bottle-fed for 1‒2 years and 364 (26%) children were bottle-fed for more than 2 years. The mean deft was lowest (1.5±0.509) in children who were not bottle-fed and highest (6.3±2.675) in children who were fed for more than 2 years. There was also a strong association between deft and use of sweetened pacifiers (P<0.001).

**Table 2:** Feeding habits, dietary habits and ECC

			**No. of children**	**%**	**Average Deft**	**Standard deviation**	**P**
**1**	**Duration of breast-feeding**	<1 year	448	32	4.65	1.996	P<0.05
		1-2 year	742	53	4.92	>2.585	
		>2 year	210	15	5.2	2.978	
**2**	**Duration of bottle-feeding**	Not Bottle fed	28	2	1.5	0.509	P<0.001
		<1 year	224	16	3.37	1.320	
		1-2 year	784	56	4.76	2.277	
		>2 year	364	26	6.3	2.675	
**3**	**Whether child fell asleep with bottle/breast-feeding at night**	Yes	1036	74	5.31	2.532	P<0.001
		No	364	26	3.65	1.862	
**4**	**Use of sweetened pacifiers**	No	1078	7	4.4	2.159	P<0.001
		Yes	322	23	6.4	2.815	
**5**	**Frequency of snacking between meals**	Seldom	56	4	2.75	1.3111	P<0.05
		Once	182	13	3.07	1.210	
		Twice	560	40	5.27	2.392	
		>3times	602	43	5.32	2.597	
**6**	**Type of snacks preferred**	Sugary	840	60	4.93	2.576	P>0.05
		Salty	560	40	4.8	2.339	

There was a significant increase in deft with frequent snacking (P<0.05) but the difference in deft between children who preferred sugary snacks than in children who preferred salty snacks was not found to be statistically significant (P>0.05; Table 2).

The average deft was lower (1.5±0.509) among the children who brushed their teeth twice daily compared to those who brushed their teeth once daily (4.3±1.012) and average deft was highest (5.42±3.048) in children who did not brush at all. However, the association between the age of commencement of tooth brushing and average deft was not found to be statistically significant (P>0.05).The education level of mother and whether both parents were working had a significant association with the severity of decay (P<0.05; Table 3).

**Table 3:** Oral hygiene habits, social factors and ECC

			**No. of children**	**%**	**Average Deft**	**Standard deviation**	**P**
**1**	**Frequency of Tooth Brushing**	No brushing	784	56	5.42	3.048	p<0.001
		Once	588	42	4.3	1.012	
		Twice	28	2	1.5	0.509	
**2**	**Age at which** **tooth brushing started**	No brush	784	56	5.4	3.048	p>0.05
		<2 year	154	11	4	1.048	
		2-3 year	238	17	4.05	1.477	
		>3 year	224	16	4.43	0.706	
**3**	**Whether the mother assisted child in tooth brushing or not**	Yes	336	24	4.20	1.259	p<0.05
		No	1064	76	5.09	2.726	
**4**	**Whether both parents were** **working outside home or not**	Yes	350	25	5.36	2.942	p<0.05
		No	1050	75	4.72	2.291	
**5**	**Educational level of mother**	Illiterate	224	16	5.87	3.245	p<0.05
		School level	504	36	5.13	2.499	
		Graduate	672	48	4.35	2.007	

## Discussion

ECC is a complex disease in which various genetic, environmental and behavioral risk factors interact. Many of these variables are highly influenced by the prevailing socioeconomic conditions, behavioral patterns and educational levels. The caries prevalence and severity cannot be determined by taking into consideration any one factor. All the factors have to be considered as a whole for health promotion so as to help promote new preventive and treatment strategies for dental caries. The term “early childhood caries” was suggested at a 1994 workshop sponsored by the Centers for Disease Control and Prevention in an attempt to focus attention on the multiple factors (i.e. socioeconomic, behavioral and psycho-social) that contribute to caries at such early ages, rather than ascribing sole causation to inappropriate feeding methods.^16^

The results of this study demonstrated that in ‘d’ component (92.6%) dominated the ‘deft’ score. The low level of dental treatment was attributed to limited accessibility to preventive and treatment services, unwillingness of practitioners to provide care for young children and primary teeth being a low priority for consideration of treatment because of a parental belief that they are temporary.

### Demographic Characteristics and ECC

The caries severity increased with age in primary dentition. This can be explained by the fact that dental caries is a cumulative process and develops over years. Thus the decayed teeth increased with age. This is in accordance with the study carried out by Hold et al (1982),^17^ Roeters et al (1995),^18^ Namal et al (2005),^19^ and Retnakumari (2012).^20^ This indicates that educational programs intended to prevent caries in deciduous teeth should begin in the first year of life among children before the condition becomes too advanced to prevent, with a view to avoiding the difficulties and huge expenses.

The mean deft was higher in children from rural areas than those from urban areas and the difference in deft was found to be statistically significant (P<0.05). This is in accordance with studies by Matilla et al (2000)^21^ and Du Minquan et al (2007).^22^ This can be explained on the basis that rural children reported less use of toothpaste or brushes, less frequent visits to a dentist and more frequent intake of sugary food. No significant association (P>0.05) was found between mean deft and the number of siblings in the present study. Namal et al (2005)^19^ also reported that the number of siblings did not increase dental caries significantly; however, there was still a slight increase.

### Feeding Habits and ECC

As this study also attempted to assess the influence of feeding habits on ECC, it was concluded that breast-feeding for longer duration increases the risk of nursing caries. Thus, although breast milk contains other immunological, nutritional and psychological advantages, prolonged breast-feeding seems to be correlated to dental diseases.^23^ Breast milk contains lactose (7%) at a higher concentration compared to bovine milk. There is decreased severity of early childhood caries with breast-feeding up to 12 months of age as compared with no breast-feeding at all. However, Albbey^24^ reported that breast-feeding, if unrestricted, results in early caries development in infants. In addition, the highest deft was recorded in children who were bottle-fed for more than 2 years. Hallet et al (2003),^25^ Mazhari et al (2007),^26^ Ekta Malvania et al (2011)^27^ and Retnakumari et al (2012)^20^ also reported increasing severity of caries with increasing duration of bottle-feeding.

In this study it was observed that 74% of children were bottle/breast-fed over night and night feeding habit was significantly associated with much more caries experience. This finding can be attributed to the fact that there is less salivary flow at night and hence less capacity for neutralization. This causes stagnation of milk in the mouth for longer periods of time and prolonged exposure of teeth to fermentable carbohydrates. The conclusion of the present finding supports AAPD statement that ‘ad libitum nocturnal breast-feeding should be avoided after the primary teeth begin to erupt’.

### Dietary Habits and ECC

The frequent intake of sweet snacks increased the risk of developing dental caries in young children, supporting nutritional recommendation of “limiting snacking times in children and encouraging regular meals but the association between type of snacks preferred and caries severity was not found to be statistically significant (P>0.05). This is consistent with a study by Maciel et al (2001),^14^ who also reported no statistically significant relationship between preference for sweets and dental caries in 4-5-year-old Brazilian children.

### Brushing Habits and ECC

In this study 56% of children did not brush at all while 42% brushed once daily. The average deft decreased as the brushing frequency increased and deft was lowest in children who brushed twice daily. This is in contrast with studies by Febres et al (1997)^28^ and Milgrom et al (2000),^29^ who failed to find any relationship between tooth brushing frequency and caries. This study supports that children who started brushing late had higher prevalence of early childhood caries so tooth brushing should be instituted when the first primary tooth erupts. Parents are models for their children and the establishment of healthy habits in their children is an important strategy for health promotion of their children. The lowest deft was found in subjects who had the supervision or assistance of an adult in brushing their teeth.

### Social Factors and ECC

Mother’s educational level also proved to be a significant predictor for caries experience in the current study. Since mothers have a major influence on the oral health behavior (feeding practices, dietary habits, food choices and tooth brushing) of their children, lack of education and information potentially influenced the child’s health status. Szatko and co-workers^30^ also found a strong interdependence on the mother’s level of knowledge with that of her educational level which influenced the child’s oral health.

## Conclusion

ECC cannot be considered only an infectious dental disease and multiple factors are involved in its etiology. It would be advisable to reach these children in time and to inform the parents about the possible causes of ECC. Thus, substantial efforts are required for early detection and treatment, and effective preventive strategies should be implemented to decrease the prevalence of ECC in preschool children.

Additional file 1. The questionnaire used for the study. This material is available online as a Word 97-2003 Document.
